# Validation of an instrument for dentists' perception of pain in patients with communication difficulties

**DOI:** 10.1590/1414-431X2023e12996

**Published:** 2023-10-20

**Authors:** M.G. Tavares, A.A. de Lima, E.N. Lia

**Affiliations:** 1 Faculdade de Ciências da Saúde, Universidade de Brasília, Brasília, DF, Brasil

**Keywords:** Validation study, Surveys and questionnaires, Pain perception, Dentists, Nonverbal communication

## Abstract

Pain is present in the dental clinic, whether due to oral problems such as dental caries and its complications or related to dental procedures. Pain evaluation in patients with communication difficulties (PCDs) is challenging for dentists, potentially compromising treatment. The aim of this study was to develop and validate an instrument to assess the perception of dentists about pain in PCDs. This study followed a quantitative methodological approach involving constructing and validating an instrument administered to 50 dentists. The initial instrument consisted of 29 items divided into four domains. Content and construct validity and internal consistency were confirmed. Content validation was performed by judges using the Content Validity Index. The instrument underwent construct validation and internal consistency assessments through exploratory factor analysis and confirmatory factor analysis using Cronbach's α, Kaiser-Meyer-Olkin, and Bartlett's sphericity tests. The final instrument consisted of 21 items divided into three domains, with a high Cronbach's α for one domain and moderate values for the others. The total variance accounted for was above 46.03%. Each factor retained at least three items, with factor loadings greater than 0.3, commonalities greater than 0.2, and eigenvalues >1. Despite the study's limitations, the instrument demonstrated its applicability and potential in evaluating the perception and management of pain in PCDs.

## Introduction

Pain is a pervasive aspect of the dental clinic, arising from various oral conditions such as dental caries and their associated complications and dental procedures. Undoubtedly, accurate assessment of pain is crucial for effective treatment. Consequently, dentists must possess knowledge and comprehension of pain to deliver appropriate care tailored to the needs of their patients (1).

However, in the context of dental practice, it is challenging to assess pain in patients with communication difficulties (PCDs), including infants, young children, people with cognitive impairment, older people with dementia, hospitalized individuals, and children with autism spectrum disorders, among others (2-4). Moreover, the COVID-19 pandemic has exacerbated communication difficulties due to widespread use of face masks and the need for physical and social distancing (5). While assessment tools are available for physicians and nurses to evaluate pain in these patients (2-4,6), their applicability in dentistry is not always straightforward. Additionally, dentists do not typically receive specific training on the assessment of pain in PCDs during their professional education (1).

The scientific literature highlights inadequate knowledge and self-awareness regarding pain assessment among dental students (1,7,8) and dentists (9,10). This deficiency has significant consequences, including under- and overtreatment of pain (3,11,12), which can contribute to various behavioral and physical disorders, cognitive decline, hospitalization, institutionalization, disability, and even premature death (13).

Therefore, the objective of this study was to develop and validate an instrument for evaluating dentists' perception of pain in PCDs.

## Material and Methods

This study obtained approval from the Research Ethics Committee of the School of Health Sciences of the University of Brasília (CAAE 30143020.4.0000.0030, opinion number 4.276.846), and the study adhered to the COSMIN recommendation checklist (14), which ensures methodological standardization in the design of questionnaires for quantitative studies.

### Study design and location

This quantitative study focused on the construction and validation of an instrument developed at the School of Health Sciences of the University of Brasília and in collaboration with the Health Secretary of the Federal District (HS-DF), Brazil.

### Development, construction, and validity of the instrument

*Step 1.* The initial domain and item generation phase involved a comprehensive literature review and extensive discussions among the research team.

*Step 2*. The instrument developed in Step 1 underwent qualitative evaluation by a panel of five expert judges. The preliminary version, created using Google Forms, was shared with the judges via a mobile message application with instructions for completion and the free and informed consent form. The data collected through Google Forms was subsequently transferred to a spreadsheet. The judges rated the items according to relevance and clarity and added comments and suggestions.

The content validity index (CVI) (15) was calculated for each item based on the assessments of the five judges. To determine the CVI, the judges rated each item by selecting one of four options: “highly relevant”, “quite relevant”, “little relevant”, or “not relevant”. The final CVI was calculated by dividing the number of judges rating an item as “highly relevant” by the total number of judges. Items with a CVI equal to or greater than 80% were considered acceptable, items with a CVI of less than 80% but equal to or greater than 50% were revised, and items with a CVI of less than 50% were discarded.

The comments provided by the judges were used for restructuring the items, reaching a consensus with the research team, and preparing the preliminary instrument for application in Step 3. The forms were distributed and subsequently returned in May 2020.

*Step 3*. In this step, ten dentists were invited to participate, representing 20% of 50 professionals from the HS-DF who provided dental care to PCDs. A mobile message containing the access link to the instrument, created using Google Forms, instructions for its completion, and the consent form was sent to the dentists. The data collected through Google Forms was then transferred to a spreadsheet for further analysis. After completing the instrument, the participants were contacted to address any doubts or clarify any aspects of the questionnaire during completion. This step was carried out in September 2020.

The adjustment made to the instrument was the reversal of the meaning of two items. The revised instrument was subsequently used in Step 4.

*Step 4*. The instrument was applied to 50 dentists from the HS-DF who provided dental care to PCDs. The process of sending the instrument and collecting data followed the previously described steps. This step took place between April and July 2021.

### Sample

For step 2, five expert judges from various health fields were selected to participate in the study based on their expertise and performance profiles. The inclusion criteria were: specialists in any health field attending or involved in undergraduate or graduate education related to care of babies, children, people with disabilities, or hospitalized individuals who cannot communicate. Exclusion criteria were: retired or outside the area of expertise.

For steps 3 and 4, a total of 50 dentists working within the Brazilian Unified Health System (SUS) in the care of PCDs were invited to participate in the research. The exclusion criterion for participants in this step was the absence from public service for more than one year.

### Statistical analysis

To assess the instrument's internal consistency, the correlation between each item and the total score of the instrument were calculated. This analysis helps determine the extent to which each item contributes to the overall measurement of the construct being assessed by the instrument.

The polychoric correlation was utilized to calculate the correlation between categorical (ordinal) variables. The variables were converted into a numerical vector based on a 5-point Likert scale: “fully disagree”, “partially disagree”, “neither agree nor disagree”, “partially agree”, and “fully agree”. The numerical vector ranged from 1 (“fully disagree“) to 5 (“fully agree“).

To establish the instrument's construct validity, the data collected with the final version of the instrument underwent exploratory factor analysis (EFA) and confirmatory factor analysis (CFA). The reliability of the instrument was assessed using Cronbach's alpha coefficient. Items with high correlation (≥0.6) (16) were kept in the instrument. The decision to retain or not retain an item in the instrument was also based on the α coefficient, estimating whether the occasional exclusion of that item increased the α value. Additionally, the Kaiser-Meyer-Olkin (KMO) (17) and Bartlett's (18) sphericity tests were conducted to evaluate the occurrence of linear relationships between variables and determine the suitability of performing a principal component analysis. Data analysis was conducted using the Statistical Package for the Social Sciences program (IBM SPSS Statistics 24, USA). The instrument's interpretability was assessed by examining the floor and ceiling effect (19). A floor effect occurs when a significant proportion (estimated at 15%) of research participants achieve the lowest score. At the same time, a ceiling effect occurs when a significant proportion of participants achieve the highest score for each domain. The lowest and highest scores for domain A are 8 and 40 points, for domain B are 7 and 35 points, and for domain C are 6 and 30 points.

## Results

### Construction of the instrument

The initial version of the instrument consisted of 29 items divided into four domains: i) Importance of pain measurement; ii) Knowledge of ways of measuring pain; iii) Evaluation of the use of pain measurement instruments; and iv) Practices adopted in the dental clinic.

### Content validity

Five judges (3 men and 2 women) participated in Step 2, including an anesthesiologist, a palliative care physician, a dentist specializing in patients with special needs, a speech therapist specializing in gerontology, and a hospital physiotherapist. The judges had an average age of 38 years (±2 years), an average professional experience of 14 years (±4 years), and an average working time in the field of 11 years (±4 years).

All 29 items were considered relevant by the judges. However, based on their suggestions and comments, 14 items underwent semantic changes while maintaining relevance. The CVI was calculated for each item, and all items met the criterion for retention (CVI ≥80%). The CVI values and the final status of each item are shown in Table 1.

**Table 1:** Content Validity Index (CVI) and final status of each item after expert judges' assessment (n=5).

Item	CVI (%)	Status
1	100%	Maintained
2	100%	Maintained
3	100%	Maintained, semantic changes
4	100%	Maintained, semantic changes
5	100%	Maintained
6	100%	Maintained
7	100%	Maintained, semantic changes
8	100%	Maintained, semantic changes
9	100%	Maintained
10	80%	Maintained, semantic changes
11	100%	Maintained
12	100%	Maintained
13	80%	Maintained
14	100%	Maintained, semantic changes
15	100%	Maintained
16	100%	Maintained, semantic changes
17	100%	Maintained
18	100%	Maintained, semantic changes
19	80%	Maintained
20	100%	Maintained
21	100%	Maintained, semantic changes
22	100%	Maintained, semantic changes
23	100%	Maintained, semantic changes
24	100%	Maintained, semantic changes
25	80%	Maintained, semantic changes
26	100%	Maintained
27	100%	Maintained, semantic changes
28	80%	Maintained
29	100%	Maintained

### Construct validity and reliability

The characteristics of the participants of Step 3 are described in Table 2. The Cronbach's alpha coefficient calculated after applying the instrument in Step 3 was 0.66. Six items showed a negative correlation, and two were reworded to address the item. However, no items were excluded from the instrument based on these findings.

**Table 2:** Characteristics of participants of the preliminary instrument application according to gender (n=10).

	Years
Age	44±9
Women (n=8)	44±9
Men (n=2)	46±11
Working time with PCDs	11±8
Women	9±8
Men	16±9
Working time in the specialty	9±8
Women	8±8
Men	11±6

Data are reported as means±SD. PCD: patients with communication difficulties.

The characteristics of the participants of Step 4 are described in Table 3. The version of the instrument developed in Step 1 was applied to the 50 dentists in Step 4. A Cronbach's alpha coefficient of 0.138 was obtained. During the analysis, several items were excluded based on different criteria. One item was excluded because it showed “zero variation” in responses. Two items were removed as they had a correlation smaller than 0.3 (r<0.3) with the other variables. Additionally, four items were removed due to the reversal effect, as they presented a correlation greater than or equal to 0.4 (r≥0.4). One item was removed as it did not show a correlation with any other item after definition of the three domains. In total, eight items were excluded in Step 4. The final KMO measure was 0.553, indicating moderate sampling adequacy, and Bartlett's Sphericity test yielded a statistically significant result (P<0.005), indicating linear relationships between variables (20).

**Table 3:** Characteristics of participants of the final instrument application according to gender (n=50).

	Years
Age	39±10
Women (n=39)	39±9
Men (n=11)	40±12
Working time with PCDs	10±8
Women	12±10
Men	10±8
Working time in the specialty	11±8
Women	11±7
Men	11±8

Data are reported as means±SD. PCD: patients with communication difficulties.

The 21 resulting items were regrouped into 3 domains (Table 4) according to the scree plot test (21) and the semantic interpretation of the authors: A) Dentist self-knowledge and knowledge of pain assessment methods (α=0.767); B) Dentist's perception of the importance of using scales (α=0.427), and C) Dentist's conduct when faced with pain by the patient (α=0.405). The total variance explained by the three domains was above 46.03%. Each factor retained at least three items, with factor loadings higher than 0.3, commonalities greater than 0.2, and eigenvalues higher than 1 (Table 4). The items included in the final instrument and the frequency of participants' responses in Step 4 are presented in Supplementary Table S1.

**Table 4:** Communalities, eigenvalues, and cumulative variance of the final instrument application (n=50).

	Domain A	Domain B	Domain C	Communalities
Eigenvalues	3.857	3.385	2.426	
Cumulative variance (%)	18.366	34.484	46.034	
Q1	**0.760**	0.286	0.002	0.659
Q2	**0.699**	-0.095	0.038	0.500
Q3	**0.621**	0.514	0.012	0.650
Q4	**0.618**	-0.004	0.013	0.382
Q5	**0.553**	-0.253	-0.069	0.375
Q6	**0.537**	0.331	0.066	0.403
Q7	**0.427**	0.156	-0.100	0.217
Q8	**0.415**	0.338	-0.350	0.409
Q9	-0.072	**0.750**	0.134	0.585
Q10	-0.313	**0.670**	0.282	0.626
Q11	0.331	**-0.616**	0.169	0.518
Q12	0.388	**0.574**	0.043	0.482
Q13	-0.500	**0.545**	-0.018	0.547
Q14	0.220	**-0.520**	0.038	0.320
Q15	-0.410	**0.453**	0.220	0.422
Q16	-0.016	0.068	**0.717**	0.519
Q17	0.274	-0.052	**0.652**	0.503
Q18	0.303	0.213	**0.632**	0.537
Q19	-0.150	-0.161	**0.584**	0.389
Q20	0.101	-0.261	**0.533**	0.363
Q21	0.211	0.279	**-0.375**	0.263

See Supplementary Table S1 for questions Q1-Q21.

### Interpretability

Interpretability, explored by the floor and ceiling effects, showed no significant responses, as no participant scored the lowest or highest in each domain.

### Hypothesis testing

Factor analysis and Cronbach's alpha showed a statistically significant (P<0.05) correlation between the items (20), although some items had low reliability (16). The observed correlation between participants' scores was consistent with the expected correlation (>0.70) in 11 of the 21 items evaluated.

## Discussion

The reliability analysis of the final instrument provided Cronbach's alpha values indicating the accuracy of the measurement as intended (22).

Results from measuring tools can be used to assess health needs, monitor effects of interventions, produce scientific evidence, identify and correct problems, provide feedback to health teams and managers, improve patient care (23), and support the development of policies and sectoral programs and the dissemination of their results (24).

Hartz (25) highlights the importance of having relevant indicators for the evaluation of health services. Despite conceptual disagreements, the adequate construction of tools for health service assessment depends on clear principles, objectives, and goals of the system to be evaluated. This clarity helps in the selection of the dimensions to be evaluated (26). In studies validating instruments for assessing health services and psychometric indicators, reliability analysis, EFA, and CFA have been commonly employed (27-30), ensuring robust and accurate tools for health service assessments.

In our study, two tests were conducted to assess the instrument's validity and reliability. The first test was factor analysis, which examined the correlation between items and determined whether they could be grouped into domains. The second test was Cronbach's alpha, which assessed the internal consistency and reliability of the items within each domain. We conducted EFA to identify the underlying structure and extract key factors or domains to evaluate the instrument's validity. CFA was then conducted to test the preconceived hypotheses about data structure identified in the EFA (29,31). EFA assumes that variables can be grouped according to their correlations, obtaining domains with all variables highly correlated with each other but have low correlations with variables outside the domain (32,33). However, EFA assumes that the variables are continuous and quantitative, using Gaussian correlation matrices for factor estimation and the exploratory factorial model. Since the variables are dichotomous, tetrachoric correlation matrices, which are appropriate for the metric of dichotomous variables, were used to estimate the factors and the factorial model (31). This approach allowed us to examine the underlying dimensions or factors of the instrument and assess its validity by confirming whether the observed data matched the hypothesized factor structure.

Our study used a five-point Likert scale to evaluate the participating professionals. This scale was chosen because it provides better reliability and validity than three-point scales. The five-point scale gives us a more nuanced measurement of opinions and attitudes and a balance between accuracy and ease of use. It is a widely used scale in research and is considered more practical and efficient than a seven-point scale. Furthermore, using a five-point Likert scale ensures accurate measurement of participant opinions while maintaining a user-friendly data collection process (34).

The initial choice of the studied domains was based on the logical sequence of learning about PCDs assistance, according to the authors of this study. The literature is consensual in suggesting a level of 60% as the minimum acceptable variance explained to define the number of domains in an instrument. However, Peterson (35) carried out a study to evaluate the levels of variance explained in studies that used factorial analysis, concluding that 10% have results lower than 34%. In our research, five domains would be obtained for a minimum explained variance of 60%, 2 of which would have only 1 item each. Indeed, the three domains obtained in the final instrument can be considered relevant as they address the essential topics related to professionals assisting PCDs. Despite a different distribution from the initial proposal, the final domains still covered the identified fundamental aspects. The application of the instrument to measure the attitudes of HS-DF professionals in assisting individuals with disabilities (PCDs) can provide valuable insights into their behaviors. By studying attitudes, which are behavior indicators, the data can help identify trends and patterns in how professionals approach and assist PCDs. This information can be used to develop targeted improvement programs and training initiatives to enhance the quality of care provided to PCDs within the HS-DF. Addressing professionals' attitudes can positively change their behaviors and improve overall support for PCDs (36).

One of the consequences of applying factor analysis was the change in the quantity and semantics of the domains developed in the preliminary instrument, as reported by Colares et al. (37). Initially, the rotated component matrix suggested the formation of 8 domains, but this number was considered excessive by the team. After the analysis, only 3 domains were adapted to the semantic characteristics of the instrument. Deleting eight items and relocating the remaining items to new domains resulted in higher factor loadings and thus greater consistency within them.

After applying the instrument in Step 4, Cronbach's alpha result was considered “slight” (22), so the items were relocated into new domains in which they presented the highest correlation, and the KMO and Bartlett's sphericity tests (18) were used to adapt it. Following the application of tests, additional items were excluded. After these changes, Cronbach's alpha was considered “substantial” for domain A and “moderate” for the other domains (22), and the final instrument was established. For this reason, the Cronbach's alpha values obtained in this validity were considered satisfactory for the purposes for which the instrument was intended (38). Thus, it is predictable that, in contexts with similar characteristics, the instrument can be used to survey the attitudes of HD-FD professionals regarding the care of PCDs.

The KMO (17) and Bartlett's sphericity tests (18) were used to assess the suitability of the data for EFA use. The latter examined whether the population correlation matrix was an identity matrix, i.e., that there was no correlation between variables with statistical significance P<0.005. Although the KMO result in our study was low (17) after eliminating the eight items, the analysis results through Bartlett's sphericity test were statistically significant, showing that the data were adequate for the principal component analysis.

In this study, criterion validity and responsiveness were not evaluated due to the absence of a “gold standard” instrument for comparison and the limited scope of the validation process, which did not allow for the assessment of changes over time. The focus of the study was primarily on aspects such as content validity, construct validity, and internal consistency of the proposed instrument. The proposed instrument was validated in a single application, making it impossible to assess construct changes over time (Responsiveness) (14).

To assess pain in PCDs, physiological indicators, such as heart rate, blood pressure, respiratory rate, or even methods that evaluate changes in skin electrical resistance can be used (3), as well as the use of one-dimensional scales that assess different behavioral responses associated with pain in cognitively impaired individuals (4). Dentists that treat PCDs must be aware of these pain assessment methods to establish the best treatment for their patients (11,12). In their study evaluating communication difficulties of hospitalized deaf patients, Sirch et al. (39) suggested that all health professionals in clinical practice should be trained to develop technical skills for proper care of these patients, either in undergraduate courses or in advanced teaching programs. In the developed instrument, domains A and B assessed the self-knowledge of the dentists and their knowledge of the existence and importance of using scales for pain assessment.

Domain C evaluated the correlation between pain measurement and professional conduct regarding this assessment. The pillars for correct pain treatment include its effective assessment and measurement (3), which directly impact the approach to patients, especially those with communication difficulties, due to inefficiency or absence of self-report.

A limitation to be considered is that there are no studies with the same objective that would be comparable to the results of this study. Furthermore, the sample size used was below that recommended in the literature [not less than 50 participants and at least five participants per item of the original instrument to be validated, that is, 145 participants according to Hair (38)]. In our study, only 50 participants were invited, as they added up to the total number of professionals who met the research inclusion criteria.

Considering the study's limitations, the instrument was shown to be feasible and to have potential to evaluate dentists' perception and management of pain in PCDs.

## Supplementary Material

Click to view [pdf].
